# The influence of pride emotion on executive function: Evidence from ERP

**DOI:** 10.1002/brb3.2678

**Published:** 2022-07-15

**Authors:** Xiao Yan Bi, Xie Ma, Aikeliya Abulaiti, Juan Yang, Yun Tao

**Affiliations:** ^1^ Faculty of Education Yunnan Normal University Kunming China

**Keywords:** Pride, cognitive flexibility, inhibitory control, working memory updating, ERPs

## Abstract

**Background:**

The current study examined the influence of positive “basic” emotions on executive function; there is limited evidence about the influence of positive “self‐conscious”emotions, such as pride, on executive functions processes.

**Methods:**

Pride is a status‐related self‐conscious emotion and the present research explored the influence of pride on the subcomponents of executive function, using three experiments that adopted the digit size‐parity switching, N‐back, and dual choice oddball paradigms.

**Results:**

The behavioral results suggested that cognitive load and behavior inhibition effects in the pride emotion were significantly higher than the neutral emotion. The ERP results showed that the pride emotion elicited smaller P3 difference wave for the switching task and dual choice oddball task. In the N‐back task, the pride emotion elicited larger N1 amplitude and smaller P2 difference wave compared to the neutral emotion. A comparison among results from the three experiments indicated that pride emotion restrains all subcomponents of executive function, though with different manifestations of the impact.

**Conclusion:**

Experiencing positive emotions is typically viewed as desirable and adaptive in educational settings; however, pride as a unique positive emotion may damage people's cognitive performance, indicating that we need to be cautious when performing cognitive operations in a pride mood.

## 1 INTRODUCTION

Pride, which refers to a self‐conscious emotion that arises from positive evaluation of oneself, and mainly depends on self‐awareness, self‐evaluation, and self‐reflection, has been extensively studied in the field of social psychology in recent years (Haidt, 2003; Lewis, 1995; Miceli et al., 2017). The positive feeling of our global “self” reinforces us to repeat the behaviors which lead us to feel proud and motivates us to pursue higher achievements; in the long term, it increases our self‐worth, and while in social interaction, the nonverbal expression of pride signifies success before other people in society and promotes social status (Heckel & Ringeisen, 2019; Tracy & Robins, 2004). The majority of previous studies focused on pride is associated with better cognitive performance (Ding, 2018; Ho et al., 2016; Kim et al., 2018; Kravchenko, 2017; Sanders et al., 2018). For example, pride has been implicated in promoting self‐control and self‐regulation, increasing cognitive flexibility and morally rational decision‐making behavior, as well as improvement of individual attention and memory retrieval capabilities. However, recent studies have shown that pride may have an inhibitory effect on cognitive performance. For example, pride was found to have impaired children's ability to delay gratification compared to joy (Shimoni et al., 2016), while those with pride showed an impaired working memory relative to those with neutral emotions (Tangney et al., 2004). There is need for an in‐depth exploration on whether pride promotes or inhibits people's cognitive behavior.

Executive functions are the attention‐regulation skills that make it possible to sustain attention, keep goals and information in mind, refrain from responding immediately, resist distraction, tolerate frustration, consider the consequences of different behaviors, reflect on past experiences, and plan for the future (Chen & Li, 2005; Cristofori et al., 2019). In fact, attention can be intentionally shifted, maintained over time, and applied selectively, and so executive function is typically measured behaviorally as the three subcomponents of cognitive flexibility, working memory updating, and inhibitory control (Diamond, 2013; Jacques & Marcovitch, 2010; Meuwissen & Zelazo, 2014). *Cognitive flexibility* refers to coordination of resource competition between different schemas. When switching a task, a participant must continuously complete multiple tasks that are continuously converted. *Working memory updating* refers to stable maintenance of multiple representations in short‐term information storage and processing systems, as well as rapid refreshing of representations when specific stimuli appear. *Inhibitory control* is the process of deliberately suppressing attention (and subsequent responding) to something, such as ignoring a distraction, stopping an impulsive utterance, or overcoming a highly learned response.

Previous studies have extensively evaluated the relationship between positive emotions and executive function, and concluded that processing of positive emotions affects executive functions, such as information processing, social judgment, attention, decision‐making, problem solving, and behavior control (Fielding et al., 2020; Pessoa, 2019; Shimoni et al., 2019). To date, however, no consensus has been reached regarding this relationship. For example, some studies have showed that positive emotions can impair executive function (Domachowska et al., 2016; Sung & Yih, 2016), while others have demonstrated that positive emotions promote or have no effect on executive function (Albert et al., 2010; Grol & De Raedt, 2018; Karalunas et al., 2020). Previous functional magnetic resonance imaging (fMRI) studies consistently reported that anterior cingulate cortex (ACC), which was involved in task switching and cognitive flexibility, monitored conflict and detected errors (Silvetti et al., 2011). For example, Wang et al.’s (2017) functional magnetic resonance imaging (fMRI) studies found that positive emotions could increase the cognitive flexibility and reduce the conflict by decreasing the activation of the dorsal anterior cingulate cortex (dACC). Moreover, Yuan, Yang, et al. (2012) used ERP to study the effect of positive emotions on behavioral inhibition control, and found that an individuals under positive emotional conditions exhibited smaller N2 and higher P3 amplitude relative to those with neutral emotions.

Although the relationship between positive “basic” emotion (joy, interest, amusement) and executive functions has been widely examined in both clinical and nonclinical populations, there is limited evidence about the influence of positive “self‐conscious” emotions, such as pride, on executive functions processes. Generally, pride is believed to have an important impact on executive function compared to joy. First, when people attribute events to their own innate advantages or acquired efforts, they experience a sense of individual self‐representation, self‐awareness, and self‐evaluation, indicating that pride is experienced when more information related to “self” is involved (Caillaud et al., 2020). Secondly, individuals under pride need to invest more emotions and have a stronger emotional activation experience (Tracy & Robins, 2004), hence they may occupy and consume more cognitive resources compared to those with positive basic emotions (DaSilva et al., 2016; Pessoa & Adolphs, 2010). Moreover, pride compared with basic emotion involved more brain regions that are related to executive function. Converging neuroimaging evidence has shown that pride could probably involve the self‐referential processing (mPFC, PCC, and precuneus), reward processing (caudate, vmPFC, septum, and OFC), memory retrieval (PCC, temporal pole, parahippocampal gyrus, and inferior temporal gyrus), social cognition (right pSTS, superior temporal gyrus), affective processing (amygdala, insula and ventral striatum) and Theory of Mind (mPFC, pSTS, and temporal pole) (Ding, 2018; Hu et al., 2019; Stolz et al., 2020). Particularly, executive function is an important basis for theory of mind (Lecce et al., 2017), whereas the working memory is an important component of executive function (Diamond, 2013). Activation of these brain regions further suggests that pride is involved in more advanced and complex cognitive operations or activities, and it may have an important impact on executive functions.

A recent study adopted the switching task and recalling imagination paradigm to explore the relationship between pride and cognitive flexibility, and found a significantly longer reaction time of the switching task under the pride condition compared to happy (Katzir et al., 2015). However, the studies have a few limitations. First, the authors did not include a neutral control group, and lacked baseline comparison; hence, they could not accurately ascertain whether the effect of pride was positive or negative. Second, for the recalling success task, it was not possible to explicitly control whether participants were thinking about the pride events or something else. Third, pride generated real‐timely as an emotional response of success or compliments could be fundamentally different than reflecting on it from the narratives (Schilbach et al., 2013). In recent years, some neuroimaging studies have used simple achievement tasks to elicit pride emotion (Ding, 2018). Particularly, this method is more time‐sensitive and ecologically valid. In addition, executive function includes three components, namely inhibitory control, working memory updating, and cognitive flexibility, which present a common basis, and are relatively independent of each other, hence can jointly determine the cognitive performance of executive function (Miyake et al., 2000). However, the current research only examines the participants' switching ability, and lacks research on the other two components of executive function. In the present study, we regard executive function as a multidimensional structure; hence, we focus on whether pride affects the three subcomponents of executive function. Generally, we are guided by the following questions: Does this influence manifest itself as a stimulating or a detrimental effect? And are the effects on different ingredients the same? Based on the previous analyses, the process of activating pride involves more cognitive operations related to “self‐awareness” and other social cognitions, occupying and depleting more cognitive resources. Therefore, we hypothesize that pride may form a resource competition with executive function tasks, while its impact on the three components of executive function may be inhibited or damaged. Therefore, three experiments tested this hypothesis by having participants achieve tasks that is likely to evoke pride versus neutral condition and then perform the digit size‐parity switching, N‐back, and dual‐choice oddball tasks as a measure of executive function.

Specifically, we adopt the ERP technology, based on high time resolution, to investigate the influence of pride on executive function through three tasks of digit size‐parity switching, N‐back, and dual‐choice oddball tasks. Firstly, we perform the digit size‐parity switching task, which mainly examines cognitive flexibility (Kopp et al., 2020), and requires participants to switch between two tasks. With regards to EEG indicators, previous studies have implicated the N2 component in conflict suppression (Provost et al., 2018; Verhoef et al., 2009), and the P3 component to decision‐making tasks (Azizian et al., 2006; Gajewski & Falkenstein, 2011). Secondly, we adopt the N‐back task, which mainly examines the ability of an individual to update their working memory (Baddeley, 2000). Functionally, this task can manipulate the change in the level of working memory load by controlling the size of parameter N, while keeping other variables constant, so the aim is to compare differences in behavioral and brain function among participants under different memory load levels. With regards to ERP components, N1 has been associated with early visual attention and response to physical properties of stimuli (Herrmannv & Knight, 2001; Lin et al., 2020), whereas the P2 component has been associated with allocation of early attention resources (Meng et al., 2012; Yang et al., 2012). Finally, we perform the dual‐choice oddball task, which mainly examines the ability to inhibit control (Yuan, He, et al., 2008; Yuan, Yang, et al., 2008). Specifically, this task requires participants to quickly and accurately make different button responses. Previous studies have shown that this task mainly induces both N2 and P3 components. Particularly, the N2 component is closely related to effective attention, reaction conflict, and activity monitoring, as well as representing the conflict detection and monitoring of different stimuli (Kemp et al., 2010; Warren et al., 2011). On the other hand, the P3 component directly reflects the reaction inhibition stage in the behavior inhibition process and is also a direct indicator of inhibition of the behavior process (Nan et al., 2018).

## 2 EXPERIMENT 1

### 2.1 Participants

Twenty‐five college students (13 males and 12 females) participated in the study. The participants’ ages ranged between 18 and 26 years (mean age ± SD = 20.84 ± 2.73). All were healthy, right‐handed, and had normal or corrected to‐normal vision. These participants had no neurological disorders or psychiatric illness. All participants signed an informed consent form, prior to inclusion in the study, while all experimental procedures were carried out in accordance with the ethical principles of the Helsinki Declaration (World Health Organization, 1996). The individuals received extra course credit or reward for their participation in the study.

### 2.2 Materials and procedure

First, we used a nine‐point Likert scale to evaluate the degree of pride emotions among participants. The scale comprised pride emotions scores that ranged from 1 to 9 representing strongly disagree and strongly agree, respectively.

Second, the participants were asked to perform a simple English vocabulary test task and generate a virtual transcript. We randomly assigned the participants into two emotional groups, namely pride (*n* = 13) and neutral (*n* = 12) groups. The vocabulary test was compiled by the Eprime program, with each test involving matching of 20 randomly‐selected words of varying difficulty. The words were considered difficult for high school learners, and each test lasted about 3 min. The virtual transcript included final scores and rankings of the students alongside 19 virtual classmates. Summarily, participants in the pride group had higher scores than their virtual classmates. Notably, the neutral group was not assigned any final scores or rankings. To avoid interference from irrelevant factors, all participants were required to perform the experiments in the laboratory. Upon completion of the simple achievement task, the participants were tested again, using subjective assessment materials of pride.

Third, the participants were asked to perform a switching task. The task was divided into the repetitive and switching phases, using 8 numbers (1, 2, 3, 4, 6, 7, 8, and 9) as stimulus materials. The 8 numbers were either colored red or green, to avoid guessing the test intention. A total of 16 stimulus items were included in the test. The participants were required to respond to the keyed numbers and colors, divided into three types as follows: (1). Large/small judgment: For red‐colored numbers, the participants were asked to press the “F” or “J” keys, if the number was less or greater than 5, respectively; (2) Odd/ even judgment: For green‐colored numbers, the participants were asked to press the “F” and “J” keys for odd or even numbers, respectively; (3) Big/small‐odd/even conversion judgment: For red‐colored numbers, the participants were to make a big/small judgment, while for green‐colored numbers, they were asked to make an odd/even judgment. The first two judgments were conducted repetitively, while the third was performed in an ABAB switching pattern. Each trial included three displays (fixation, target, and blank). Firstly, the fixation was displayed at the center of the screen for 500 ms. Then the target number was presented in the center of the computer monitor for 2000 ms until the response was made. And then this target display was replaced by a blank screen. The screen remained blank for an additional 500 ms after the response, and then the target number for the next trial was presented. The experimental procedure was divided into practice and formal experimental stages, and participants were allowed to reach a practice accuracy rate of more than 80% prior to commencement of the formal experiment. The formal experiment comprised three blocks, with a total of 200 trials.

### 2.3 ERP recording and analysis

Using Brain Products (Munich, Germany), brain electrical activity was recorded from 64 Ag/AgCl electrodes mounted on an elastic cap. The data were referenced online to FCz and offline re‐referenced to the algebraic average of the left and right mastoids. An impedance of less than 5 kΩ was maintained in all electrodes. The horizontal electrooculograms and vertical electrooculograms were collected from the left against the right orbital rim and infra and supra arbitrarily at the left eye, respectively. Then continuous sampling at 1000 Hz and an FIR filter (0.01−80 Hz band filter) to amplify the signals for offline analysis were performed. ERP analysis epochs were extracted offline and included 200 ms of prestimulus activity and 1000 ms of poststimulus activity.

Based on previous studies, we set the analysis time window for N2 and P3 at 150–220 ms and 270–450 ms, respectively (Cona et al., 2015; Nedeljkovic & Kyrios, 2007). We adopted the average amplitude method to select the analysis time window of N2 and P3, two EEG components, and then selected nine electrode points as F3, FZ, F4, P3, PZ, P4, C3, CZ, and C4 for analysis. We performed emotion type (pride, neutral) × task type (repetition, switching) × electrode points (F3, FZ, F4, P3, PZ, P4, C3, CZ, C4) and repeated‐measures ANOVA was conducted. For all analyses, the degrees of freedom of the *F* ratio were corrected for violations of the sphericity assumption based on the Greenhouse Geisser correction (Greenhouse & Geisser, 1959).

### 2.4 Results

#### 2.4.1 Behavioral results

The paired‐sample *t*‐test was used to detect the effectiveness of emotion induction; it revealed that pride emotion induction led to significantly higher pride emotion (*M* = 5.92±1.55) relative to before induction (*M* = 4.38±1.56). Conversely, there were no significant differences in the neutral emotion. Overall, these findings confirmed that the simple vocabulary test task successfully induced pride emotion. The ANOVA on RT revealed that the main effect of task type was significant, *F* (1,23) = 155.43, *p* < 0.001, *η*2 p = 0.87. The reaction time of the switching task was significantly longer than the repetitive task. In addition, the main effect of emotion type was significant, *F* (1,23) = 4.52, *p* = 0.045, *η*2 p = 0.16). The reaction time of the pride emotion was significantly longer than the neutral emotion. However, the two‐way interaction was not significant, *F* (1,23) = 3.05, *p* = 0.094. The ANOVA on ACC revealed that the main effect of task type was significant, *F* (1,23) = 112.52, *p* < 0.001, *η*2 p = 0.83, with the accuracy rate of switching task being significantly lower than the repetitive task. However, the main effect of emotion type was not significant, *F* (1,23) = 0.37, *p* = 0.550. Similarly, the two‐way interaction was not significant, *F* (1,23) = 1.85, *p* = 0.187 (Table 1).

**TABLE 1:** Reaction time (ms) and accuracy (%) in different emotion types in the switching task paradigm

Type	Task repetition		Task switching	
	RT	ACC	RT	ACC
Pride	561.86(44.62)	96.23(3.00)	1045.67(142.66)	85.23(4.81)
Neutral	544.39(87.78)	95.75(1.77)	909.39(179.76)	87.25(5.08)

(RT = reaction time, ACC = accuracy rate).

#### 2.4.2 ERP results

**FIGURE 1:**
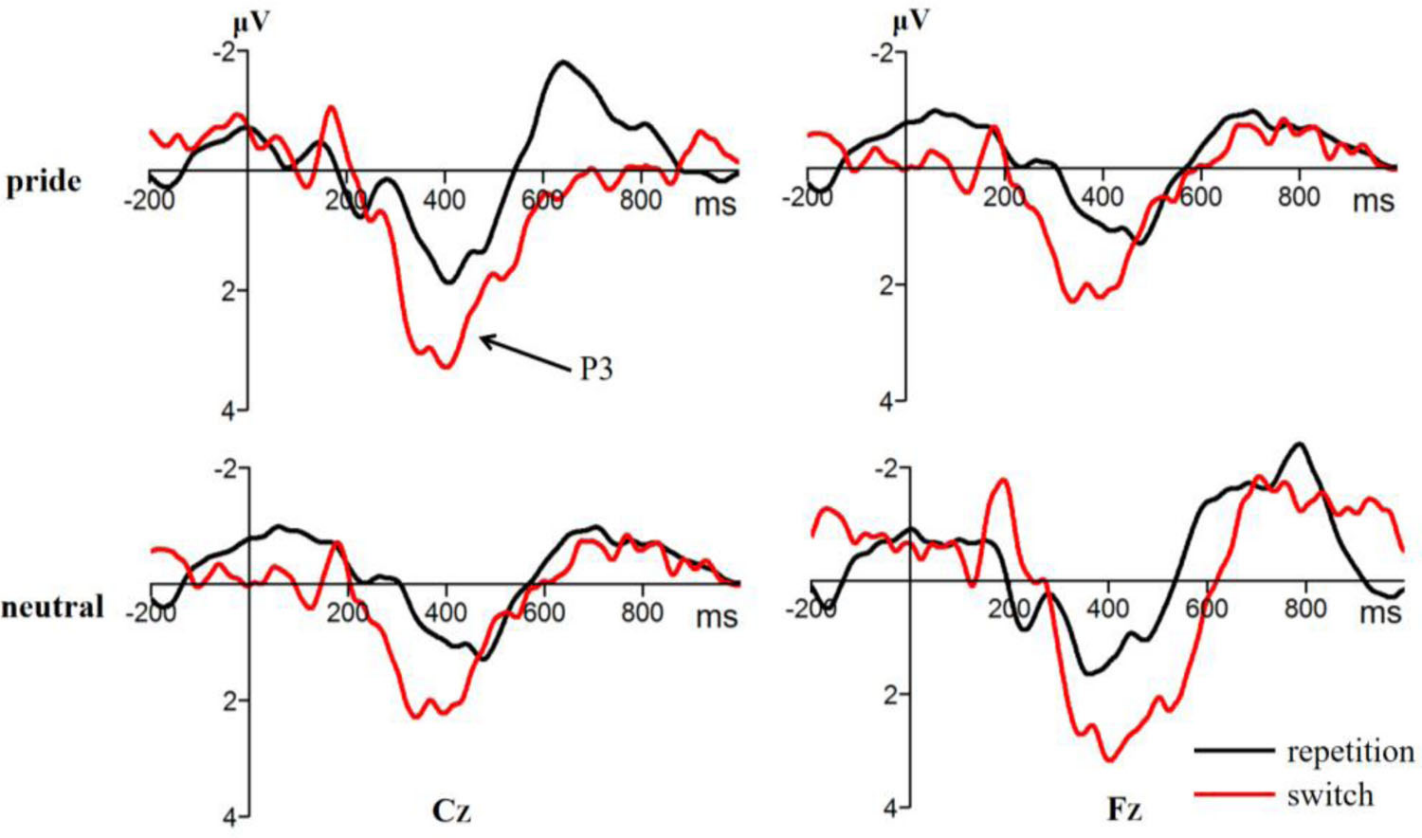
The original waveforms of the digit size‐parity switching task on the Cz and Fz electrode points under emotion conditions

**FIGURE 2:**
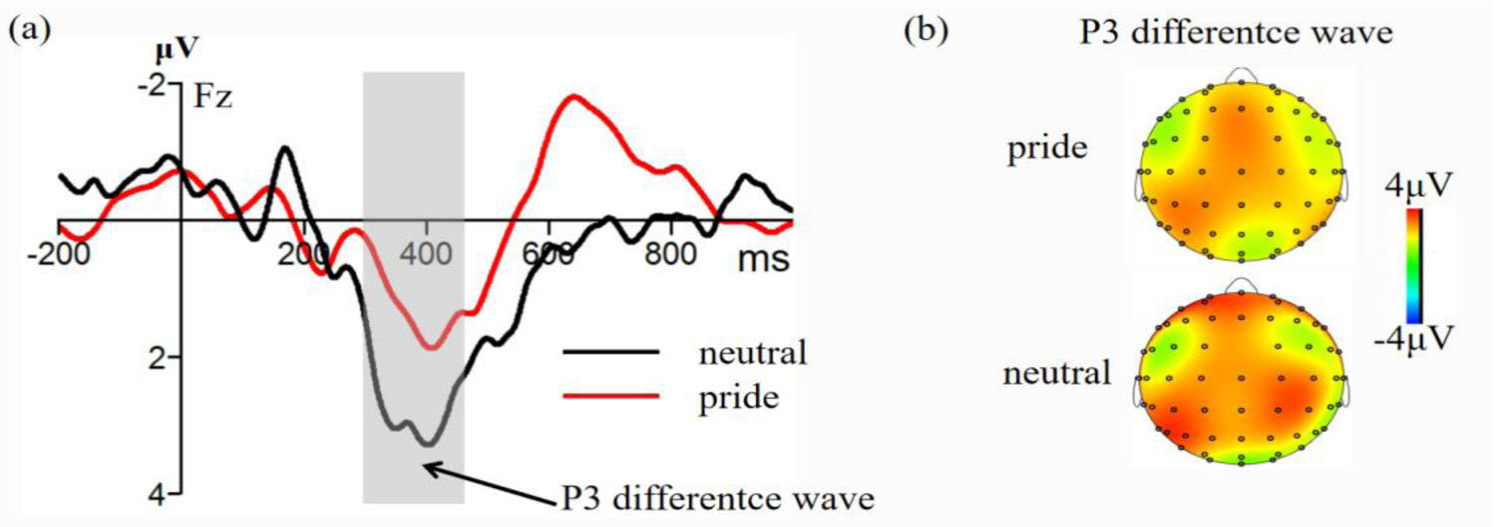
(a) Waveform of the P3 difference wave (switching tasks amplitude minus repeated tasks amplitude) on the Fz electrode point under pride and neutral emotion. (b) Topographic map showing the P3 difference wave

Figures 1 and 2 present the pooled activities of representative electrodes by condition for each ERP component. For the N2, the ANOVA only revealed an effect of electrode point, *F* (4,95) = 10.33, *p* < 0.001, *η*2 p = 0.31, but neither the effect of task type, *F* (1,23) = 0.54, *p* = 0.469, nor the emotion type, *F* (1,23) = 0.01, *p* = 0.944, was significant. Similarly, the interactions between the task type and emotion type were not significant, *F* (1,23) = 0.48, *p =* 0.497.

For the P3, the ANOVA revealed that the main effect of task type was significant, *F* (1,23) = 23.38, *p *< 0.001, *η*2 p = 0.51. The switching tasks elicited a greater P3 amplitude than the repetitive tasks. The main effect of the emotion type was not significant, *F* (1,23) = 1.77, *p =* 0.071. The main effect of the electrode point was significant, *F* (3,65) = 3.12, *p* = 0.034, *η*2 p = 0.12. In addition, the interactions between task type and emotion type were also significant, *F* (1,23) = 8.68, *p* = 0.007, *η*2 p = 0.28. Independent sample *t*‐test showed that the P3 difference wave (switching tasks amplitude minus repeated tasks amplitude) of the pride emotion was significantly higher than the neutral emotion, *t* (23) = 2.87, *p* = 0.012, 95% CI = [2.98, 20.31], Cohen's *d* = 0.77.

### 2.5 Discussion

In experiment 1, we used the digit size‐parity switching task then combined the ERP technology to reveal the effect of pride on cognitive flexibility. Behavioral results revealed that the switching task had longer reaction time and lower accuracy rates than repetitive tasks, which was consistent with previous studies (Kessler et al., 2017; Moradzadeh et al., 2015). Consistent with behavioral results, our ERP revealed that the switching task elicited a larger P3 amplitude than the repetitive task, indicating that it generates a significant switching effect on the P3 component, which was consistent with previous studies (Gajewski & Falkenstein, 2011; Kopp et al., 2020). However, the N2 component resulted in no significant switching effect. The two‐stage models assume that task preparation is not switch‐specific but occurs in both switch trials and repetition trials, which may explain the lack of significant differences between the switching and repetitive tasks (Kiesel et al., 2010).

In addition, we found that the emotional effect was not significant on the N2 component. Previous studies have shown that in the switching task paradigm, the N2 component reflects the ability to monitor conflicts (Deng et al., 2015; Gajewski et al., 2018). Results of experiment 1 showed that pride had no significant effect on conflict‐monitoring ability. In contrast, it had a significant emotional effect on the P3 component. The pride emotion inducted a significantly smaller P3 difference wave than the neutral emotion. Previous studies have shown that the P3 difference wave reflects the top‐down decision‐making process in the switching task paradigm, that is, the response to the execution process of the selection. Generally, a small P3 difference wave implies performance during the decision‐making process (Gajewski et al., 2017; Hillman et al., 2006). The findings of Gajewski and Falkenstein (2012) revealed a smaller P3 difference wave without any training group compared to training group. Moreover, the team found that elderly participants had difficulty in completing the switching task compared to younger participants, since the former group needs to put in more effort to elicit a smaller P3 difference wave (Gajewski et al., 2018). Furthermore, results from time–frequency analysis confirmed that a reduction in network power caused a subsequent reduction in P3 difference wave, indicating that the reduction in P3 difference wave is a sign of difficulty during the decision‐making process (Enriquez‐Geppert & Barceló, 2018). In summary, the small P3 difference wave may impair their decision‐making ability of pride emotion.

Based on results of this experiment, pride possibly inhibited task decision‐making ability due to the fact that an individual puts more emotions in pride emotion, which has higher task requirements and a greater cognitive load (Tracy & Robins, 2004), which cause a decrease in the P3 difference wave. In addition, pride is a process involving high self‐involvement. Therefore, under the conditions of high self‐involvement, an individual will invest more psychological resources, but exhaust them when completing the current task, which also significantly reduces the P3 difference wave (Chen et al., 2020; Zhu et al., 2016). Taken together, these findings indicate that pride inhibits cognitive flexibility.

## 3 EXPERIMENT 2

### 3.1 Participant

Twenty‐nine college students (15 males and 14 females) participated in the study. The participants’ ages ranged between 18 and 26 years (mean age ± SD = 20.76 ± 2.06). All were healthy, right‐handed, and had normal or corrected to‐normal vision. These participants had no neurological disorders or psychiatric illness. All participants signed an informed consent form, prior to inclusion in the study, while all experimental procedures were carried out in accordance with the ethical principles of the Helsinki Declaration (World Health Organization, 1996). The individuals received extra course credit or reward for their participation in the study.

### 3.2 Materials and procedure

First, we used a nine‐point Likert scale to evaluate the degree of pride emotions among participants. The scale comprised pride emotions scores that ranged from 1 to 9 representing strongly disagree and strongly agree, respectively.

Second, the participants were asked to perform a simple digital test task and generate a virtual transcript. We randomly assigned the participants into to two emotional groups, namely pride (*n* = 15) and neutral (*n* = 14) groups. The digital test task was compiled by the Eprime program. Each test randomly presents 20 numerical addition or subtraction calculation questions, the numerical difficulty in the numerical calculation questions was 2 digits, and each test lasts about 5 min. The virtual transcript comprised participants’ final scores and rankings in comparison to 19 virtual classmates. Participants in the pride group ranked in the top three across numerical tests, while those in the neutral group were not assigned final scores and rankings. Upon completion of the simple achievement tasks, the participants were tested again, using subjective assessment materials of pride.

Third, we used 26 letters as the experimental stimulus material, and two cognitive load levels (1‐back and 2‐back) of the N‐back paradigm. Specifically, this task uses the Eprime 2.0 to program the letter N‐back for working memory updating. During the 1‐back task, participants were asked to compare and indicate whether the current letter was the same as the one that had appeared before it. In the 2‐back task, they were asked to compare and indicate whether the current letter was the same as the second letter that had appeared before it. They were asked to press the F and J keys for the same and different, respectively. The ratio of target stimulus to nontarget stimulus was 1:2, and was presented in a random order. Each trial included two displays (fixation, and test). Firstly, the fixation was displayed at the center of the screen for 800 ms. Then, the test letter was presented in the center of the computer monitor for 2000 ms until the response was made, and then the fixation display for the next trial was presented. The experimental procedure was divided into practice and formal experimental stages, and participants were allowed to reach a practice accuracy rate of more than 80% prior to commencement of the formal experiment. The formal experiment comprised two blocks, with a total of 120 trials.

### 3.3 ERP recording and analysis

Based on previous studies, we set the analysis time window for N1 and P2 at 80–140 ms and 170–240 ms, respectively (Irak et al., 2020; Lin et al., 2020). We adopted the average amplitude method to select the analysis time window of N1 and P2 two EEG components, then selected nine electrode points as F3, FZ, F4, P3, PZ, P4, C3, CZ, and C4 for analysis. We performed emotion type (pride, neutral) × cognitive load (1‐back, 2‐back) × electrode points (F3, FZ, F4, P3, PZ, P4, C3, CZ, C4) repeated‐measures ANOVA. For all analyses, the degrees of freedom of the *F* ratio were corrected for violations of the sphericity assumption based on the Greenhouse Geisser correction (Greenhouse & Geisser, 1959).

### 3.4 Results

#### 3.4.1 Behavior results

The paired‐sample *t*‐test was used to detect the effectiveness of emotion induction, revealing that pride induction resulted in a significantly higher pride emotion (*M* = 5.67±1.71) relative to before induction (*M* = 4.73±1.28). Conversely, there were no significant differences in the neutral emotion. Overall, these findings confirmed that the simple digital test task successfully induced pride emotion. The ANOVA on RT revealed that the main effect of cognitive load was significant, *F* (1,27) = 89.28, *p* < 0.001, *η*2 p = 0.77. The reaction time of the 1‐back task was significantly longer than the 2‐back task. In addition, the main effect of emotion type was significant, *F* (1,27) = 22.19, *p* < 0.001, *η*2 p = 0.45. The reaction time of the pride emotion was significantly longer than the neutral emotion. However, the two‐way interaction was significant, *F* (1,27) = 10.84, *p =* 0.003, *η*2 p = 0.29. Independent sample *t*‐test showed that the cognitive load effect of the pride emotion on reaction time was significantly higher than the neutral emotion, *t* (27) = 3.35, *p* = 0.003, 95% CI = [54.18, 229.16], Cohen's *d* = 0.81. The ANOVA on ACC revealed that the main effect of cognitive load was significant, *F* (1, 27) = 41.32, *p* < 0.001, *η*2 p = 0.61. The accuracy rate of 2‐back task was significantly lower than the 1‐back task. In addition, the main effect of emotion type was significant, *F* (1, 27) = 7.17, *p* = 0.012, *η*2 p = 0.21. The accuracy rate of pride emotion was significantly lower than the neutral emotion. However, the two‐way interaction was not significant, *F* (1,27) = 7.10, *p* = 0.013, *η*2 p = 0.21. Independent sample *t*‐test showed that the cognitive load effect of the pride emotion on accuracy rate was significantly higher than the neutral emotion, *t* (27) = 2.74, *p* = 0.014, 95% CI = [0.02, 0.17], Cohen's *d *= 0.79 (**Table** **2** **)**.

**TABLE 2:** Reaction time (ms) and accuracy rate (%) of different emotion types in the N‐back task paradigm

Type	1‐back		2‐back	
	RT	ACC	RT	ACC
Pride	563.19 (100.62)	96.07 (2.46)	837.33 (128.31)	79.67 (11.95)
Neutral	448.29 (133.78)	95.93 (2.56)	580.75 (118.94)	89.14 (4.74)

#### 3.4.2 ERP results

**FIGURE 3:**
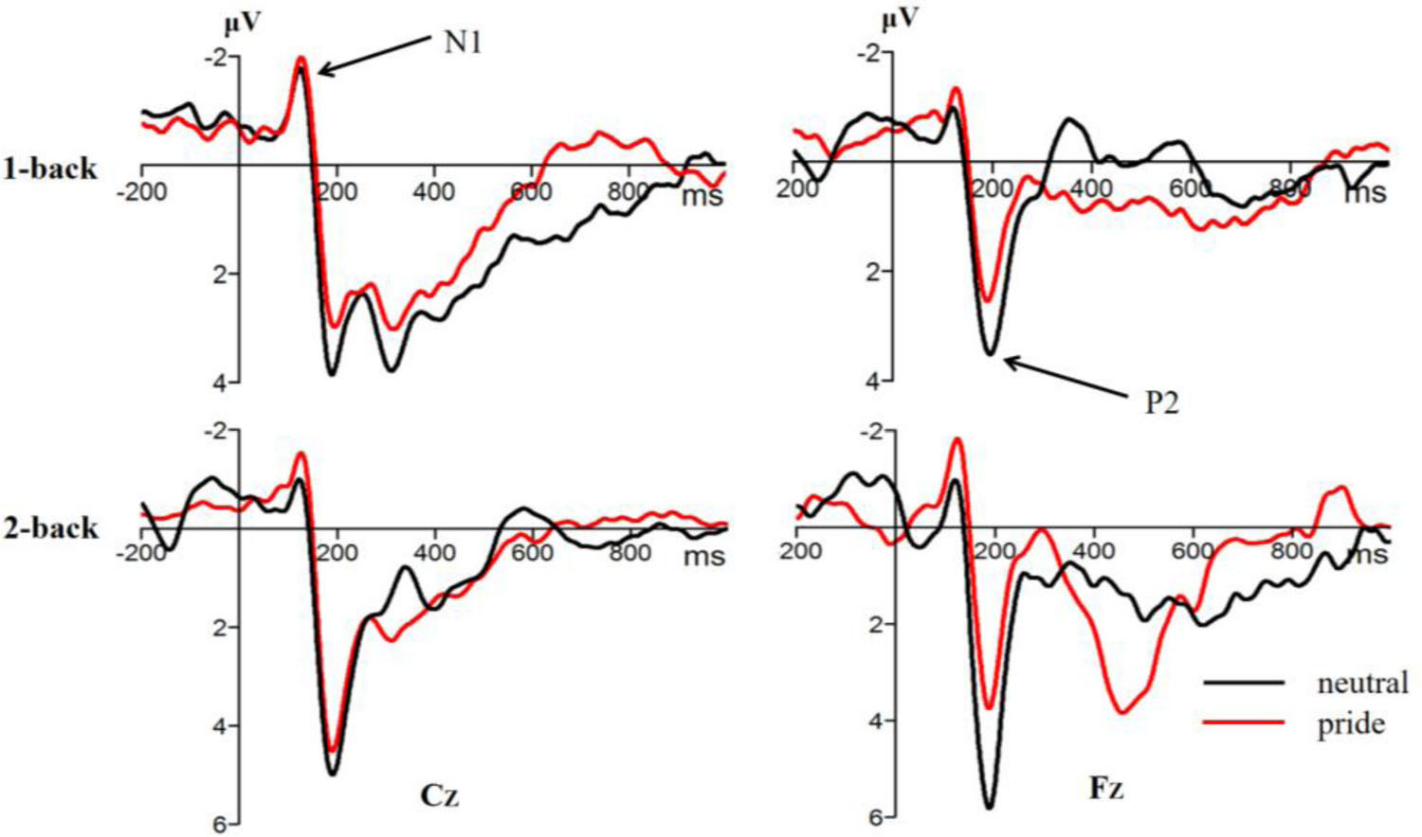
The original waveforms of pride and neutral emotions on *C*z and *F*z electrode points under different cognitive load conditions

**FIGURE 4:**
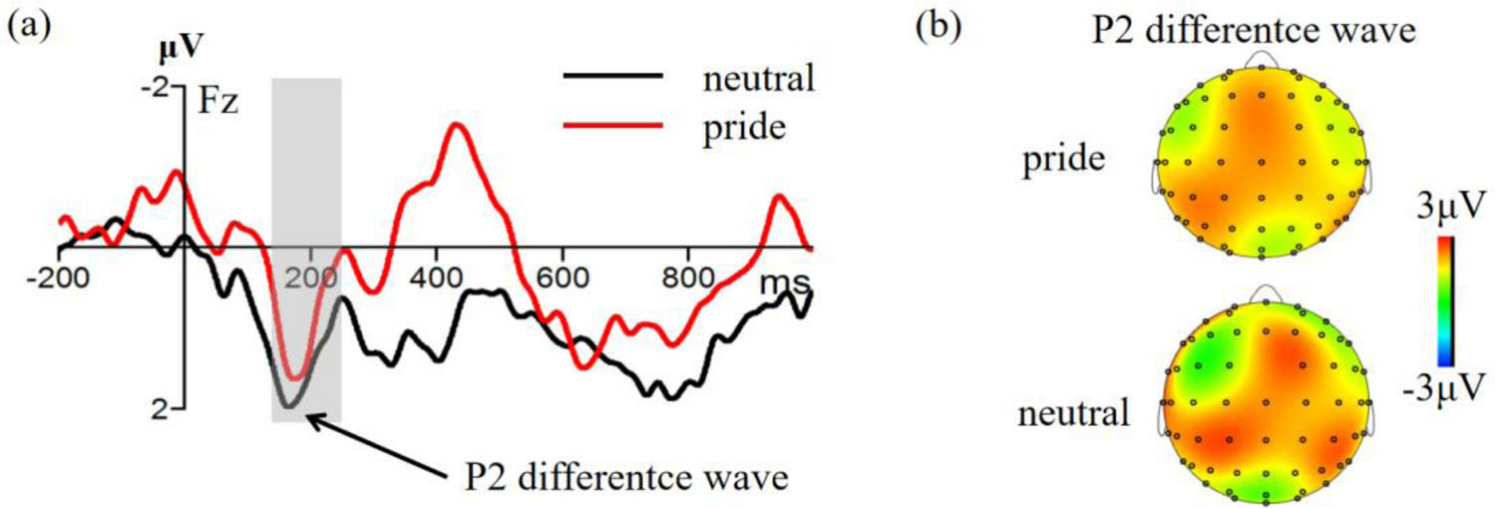
(a) Waveform of P2 difference wave (2‐back amplitude minus 1‐back amplitude) on the Fz electrode point under pride and neutral emotion; (b) Topographic map showing P2 difference wave

Figures 3 and 4 present the pooled activities of representative electrodes by condition for each ERP component. For the N1, the ANOVA only revealed an effect of emotion type, *F* (1,27) = 6.37, *p* = 0.018, *η*2 p = 0.19. The pride emotion induced a greater N1 amplitude than the neutral emotion. But neither the effect of cognitive load, *F* (1,27) = 0.01, *p =* 0.950, nor the electrode point, *F* (5,132) = 1.15, *p =* 0.338, was significant. Similarly, the interactions between the cognitive load and emotion type were not significant, *F* (1,27) = 0.97, *p =* 0.333.

For the N2, the ANOVA revealed that the main effect of cognitive load, *F* (1, 27) = 1.27, *p =* 0.269, and the effect of emotion type, *F* (1, 27) = 0.01, *p =* 0.991, were not significant. However, the main effect of the electrode point was significant, *F* (3,85) = 13.97, *p* < 0.001, *η*2 p = 0.34. Moreover, the interactions between cognitive load and emotion type were significant, *F* (1, 27) = 9.77, *p* = 0.004, *η*2 p = 0.27. Sample *t*‐test showed that the P2 difference wave (2‐back amplitude minus 1‐back amplitude) of the pride emotion was significantly smaller than the neutral emotion, *t* (27) = 3.15, *p* = 0.004, 95% CI = [10.10, 47.96], Cohen's *d* = 0.64.

### 3.5 Discussion

In experiment 2, we used the N‐back task and then combined the ERP technology to reveal the effect of pride on working memory updating. The behavioral results showed that high cognitive load has longer reaction time and lower accuracy rate compared with low cognitive load, which was consistent with the previous studies (Clark et al. al., 2017; Yaple et al., 2019). In addition, the cognitive load effect of the reaction time and accuracy rate of the pride emotion were significantly higher than in the neutral emotion, indicating that pride hindered the ability to working memory updating. Consistent with behavioral results, our ERP revealed that N1 was related to early visual attention. Notably, a larger amplitude was related to more early attention (Herrmann & Knight, 2001; Lin et al., 2020). The P2 component was mainly related to selective attention. A larger amplitude was related to more selective attention (Vilà‐Balló et al., 2018; Yuan et al., 2016). The current study revealed that the pride emotion elicited a larger N1 amplitude and a smaller P2 difference wave compared with the neutral emotion. This finding indicates that the N‐back task in the pride emotion can get more early attention, however, less selective attention occurs in the later stage, indicating that pride affects the ability of working memory updating. Several theories and opinions explain the effect of emotion on the working memory. The theory of the social function of emotions proposes that development of emotions maintains interpersonal relationships and establishes a social hierarchy (Fischer & Manstead, 2008; Keltner & Haidt, 1999). Under such conditions, individuals will continue to maintain positive experiences; thus the individual will devote more attention to completing the current task in the early stage, ultimately inducing a larger N1 amplitude in the early stage. As the number of memory tasks increases in the later selective attention, the resources allocated to each task show a decrease. Therefore, pride may compete with cognitive tasks thus making task processing difficult, and as a result induces a smaller P2 difference wave. In addition, the hindering effect of pride on working memory updating may be due to the need for a series of complex cognitive activities under the pride emotion, such as self‐conscious, self‐representation, and self‐evaluation, which affect the process of generating pride. Therefore, there is a shortage of resources for completion of subsequent cognitive tasks, thus pride hinders the ability of working memory updating.

## 4 EXPERIMENT 3

### 4.1 Participants

Twenty‐eight college students (15 males and 13 females) participated in the study. The participants’ ages ranged between 18 and 26 years (mean age ± SD = 21.00 ± 1.98). All were healthy, right‐handed, and had normal or corrected to‐normal vision. These participants had no neurological disorders or psychiatric illness. All participants signed an informed consent form, prior to inclusion in the study, while all experimental procedures were carried out accordance with the ethical principles of the Helsinki Declaration (World Health Organization, 1996). The individuals received extra course credit or reward for their participation in the study.

### 4.2 Materials and procedure

The experiment uses the vocabulary test task to induce the participants' pride emotion that was similar to experiment 1. Then, the participants were asked to perform a digital test task. The experimental procedure was as follows, a continuous gaze point is presented at the center of the computer screen for 300 ms at the beginning of each trial. After the gaze point, a random empty screen with a time between 500 and 1000 ms was presented and then the experimental stimulus was presented. Two types of stimuli were presented: ① When the standard stimulus (letter O) appeared, the participants were required to press the F key to respond as soon as possible; ② When the deviation stimulus (letter Q) appeared, participants were required to press the J key to respond as soon as possible. The stimulus presentation time limit was 1000 ms and stimulus presentation was terminated when the participants pressed the key. The gaze point “+” then appeared repeatedly for each cycle. After each block, the correct rate of the block was presented to the participants. Participants were required to complete 20 trials of practice before the formal experiment, and then begin the formal experiment after achieving a correct rate of 100%. The formal experiment contained 3 blocks, each block comprising 100 trials, including 75 standard stimuli and 25 deviation stimuli. The two stimuli were presented randomly.

### 4.3 ERP recording and analysis

Based on previous studies, we set the analysis time window for N2 and P3 at 210–270 ms and 300–450 ms, respectively (Wang et al., 2011). We adopted the average amplitude method to select the analysis time window of N2 and P3, two EEG components, and then selected nine electrode points as F3, FZ, F4, P3, PZ, P4, C3, CZ, and C4 for analysis. We performed emotion type (pride, neutral) × stimulation type (standard, deviation) × electrode points (F3, FZ, F4, P3, PZ, P4, C3, CZ, C4) repeated‐measures ANOVA. For all analyses, the degrees of freedom of the *F* ratio were corrected for violations of the sphericity assumption based on the Greenhouse Geisser correction (Greenhouse & Geisser, 1959).

### 4.4 Results

#### 4.4.1 Behavior result

The ANOVA on RT revealed a significant effect of stimulation type, *F* (1,26) = 10.78, *p =* 0.003, *η*2 p = 0.29. The reaction time of the deviation stimulation was significantly longer than the standard stimulation. However, the main effect of emotion type was not significant, *F* (1,26) = 2.48, *p =* 0.128. Similarly, the two‐way interaction was not significant, *F* (1,26) = 0.04, *p =* 0.843. The ANOVA on ACC revealed a significant effect of stimulation type, *F* (1,26) = 43.86, *p* < 0.001, *η*2 p = 0.63, where the accuracy rate of deviation stimulation was significantly lower than the standard stimulation. In addition, the main effect of emotion was not significant, *F* (1,26) = 3.23, *p* = 0.084. However, the two‐way interaction was significant, *F* (1,26) = 4.26, *p =* 0.049, η2 p = 0.14. Independent sample *t*‐test showed that the behavior inhibition effect of the pride emotion on accuracy rate was significantly higher than the neutral emotion, *t* (26) = 2.06, *p* = 0.049, 95% CI = [0.01, 0.08], Cohen's *d* = 0.76 (**Table** **3** **)**.

**TABLE 3:** Reaction time (ms) and accuracy (%) of different emotion types in the dual choice oddball paradigm

Type	Standard stimulation		Deviation stimulation	
	RT	ACC	RT	ACC
Pride	402.60 (33.79)	99.38 (1.21)	462.21 (46.87)	91.00 (7.00)
Neutral	378.55 (17.77)	99.27 (0.70)	431.32 (110.81)	94.87 (2.95)

#### 4.4.2 ERP results

**FIGURE 5:**
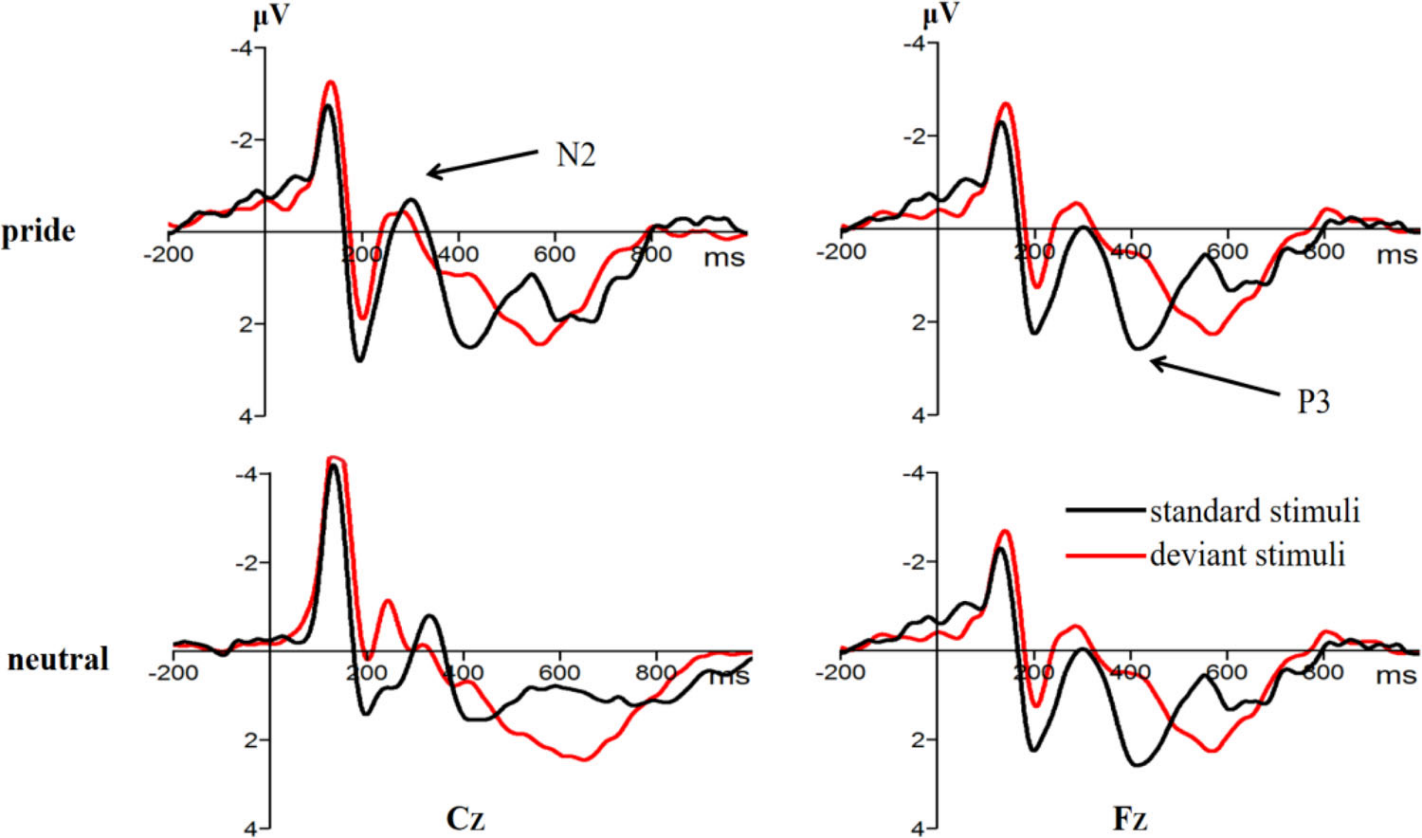
The original waveforms of the oddball task on the Cz and Fz electrode points under the pride and neutral emotion conditions

**FIGURE 6:**
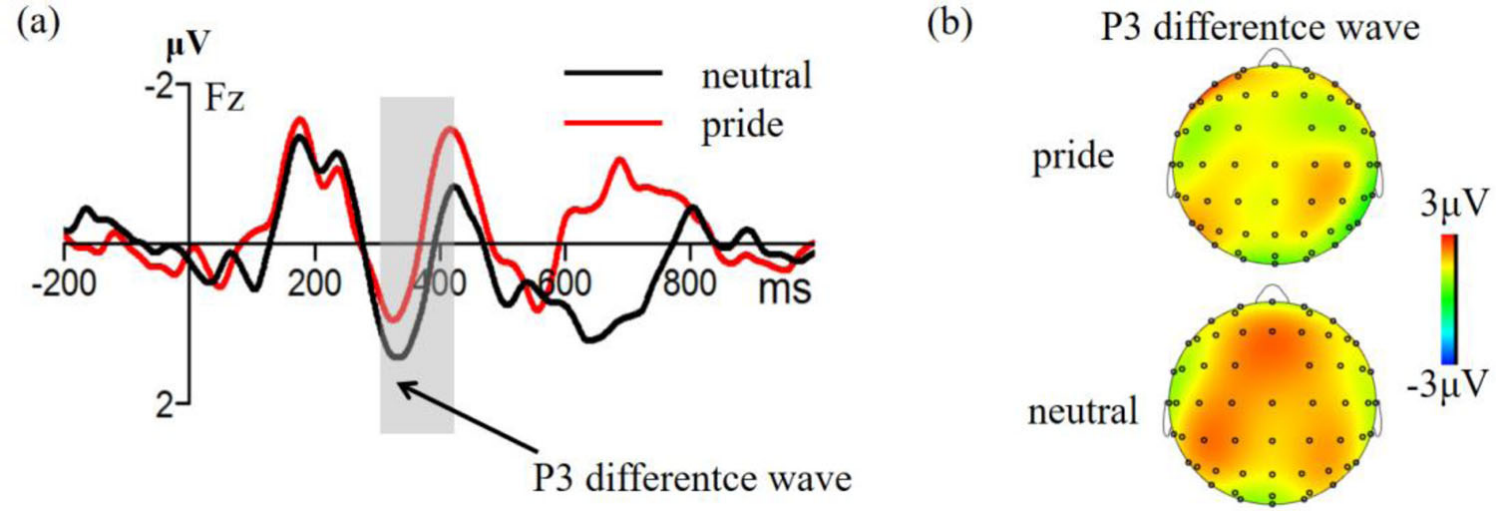
(a) Waveform of P3 difference wave (deviation stimulation amplitude minus standard stimulation amplitude) on the Fz electrode point under pride and neutral emotion; (b) Topographic map showing P2 difference wave

Figures 5 and 6 present the pooled activities of representative electrodes by condition for each ERP component. For the N2, the ANOVA revealed that the main effect of emotion type, *F* (1,26) = 5.99, *p =* 0.021, *η*2 p = 0.19, and the effect of electrode point, *F* (3,67) = 4.95, *p* < 0.001, *η*2 p = 0.16, were significant. The pride emotion induced a greater N2 amplitude than the neutral emotion. However, the main effect of the stimulation type was not significant, *F* (1,26) = 0.17, *p *= 0.682. Moreover, the interaction between stimulation type and emotion type were also not significant, *F* (1,26) = 0.12, *p =* 0.737.

For the P3, the ANOVA revealed that the main effect of stimulation type was significant, *F* (1, 26) = 26.47, *p *< 0.001, *η*2 p = 0.51. The deviation stimulation induced a greater P3 amplitude than the standard stimulation. In addition, the main effect of emotion type was significant, *F* (1, 26) = 9.74, *p =* 0.004, *η*2 p = 0.27. The pride emotion induced a greater P3 amplitude than the neutral emotion. However, the effect of electrode point was not significant, *F* (3, 64) = 1.95, *p =* 0.142. Notably, the interactions between stimulation type and emotion type were significant, *F* (1, 26) = 8.79, *p* = 0.006, *η*2 p = 0.26. Independent sample *t*‐test showed that the P3 difference wave (deviation stimulation amplitude minus standard stimulation amplitude) of the pride emotion was significantly smaller than the neutral emotion, *t* (26) = 2.96, *p *= 0.006, 95% CI = [5.04, 27.87], Cohen's *d* = 0.59.

### 4.5 Discussion

We used the dual‐choice oddball task and then combined the ERP technology to reveal the effect of pride on inhibitory control in experiment 3. The behavioral results showed that deviation stimulus had longer reaction time and lower accuracy rate compared with the standard stimulus, which was consistent with the previous studies (Fogarty et al., 2019; Yuan et al., 2012). In addition, behavioral inhibition effect on the accuracy rate of the pride emotion was significantly higher than the neutral emotion, indicating that pride emotion interfered with the individual's inhibitions. Consistent with behavioral results, our ERP showed that the deviation stimulus induced a larger P3 amplitude than the standard stimulus, indicating that this paradigm can induce a significant behavioral inhibitory effect, which was consistent with previous studies (Wang & Dai, 2020; Yuan, He, et al., 2008; Zhou et al., 2020). In addition, the findings showed that the pride emotion induced a smaller P3 difference wave compared with the neutral emotion. However, the effect on N2 amplitude was not significant. Previous studies explored P3 difference wave as an indicator of behavioral inhibition. The change in P3 difference wave may indicate the ability of participants to successfully suppress conflict. A larger P3 difference wave is correlated with a stronger inhibition ability (Chen et al., 2008; Wessel, 2018). In the current study, the pride emotion induced a smaller P3 difference wave, indicating that the individual's inhibitory control was weakened under the condition of pride. This implies that pride was involved in the self, and self‐related individuals had a strong desire to maintain a sense of superiority (Tangney et al., 2007), resulting in the individual consuming part of the psychological resources in the process. Therefore, relatively few cognitive resources were used to process new tasks indicating that the inhibitory ability was damaged resulting in reduction of P3 difference wave. Previous studies report that emotions can interfere with inhibitory control. Mikheenko (2013) used the Go/Nogo task and reported that it was challenging for participants to inhibit unsuitable reactions and the findings showed a higher rate of false reports under positive emotional conditions. Stadler et al. (2007) reported that children with behavioral control disorders exhibited significantly low activation of the right ACC responses to emotional pictures indicating that behavioral control disorders may be correlated with abnormal emotional activities. In summary, the findings from experiment 3 showed that pride had interference effect on inhibitory control. This interference effect may be correlated with the middle and late inhibitory control. However, it did not have a significant effect on the N2 conflict‐related effects.

## 5 GENERAL DISCUSSION

Three experiments were conducted in the current study to explore the effect of pride emotion on the components of executive function including cognitive flexibility, working memory updating, and inhibitory control. Achievement tasks were used to induce pride in the three experiments, and two induction methods were used in this study to avoid repetition. The findings showed that both the vocabulary test task and the number test task effectively induced pride emotion.

Analysis of ERP showed that pride emotion elicits a smaller P2 amplitude in the working memory updating (N‐back task); however, the findings showed no significant difference in cognitive flexibility and inhibitory control. This finding indicates that pride affects only the early stage of working memory updating, which may be related to the individual's selective attention. In the N‐back task, the individual needs to pay attention to the current stimulus and previous or the last two times stimulus, thus the individual's attention needs to be allocated. In addition, under the influence of the pride emotion, individuals need to devote a certain amount of attention resources to events that make the individual proud. In this case, there are fewer attention resources for the task at hand, resulting in a smaller P2 difference wave.

Analysis of the P3 component showed that the pride emotion elicited a smaller P3 difference wave in the switching task and the inhibitory control task. This indicates that pride emotion has an effect on cognitive flexibility and inhibitory control. However, the effects on the two tasks were different. The switching task may mainly involve the task decision‐making process, whereas the inhibitory control task may mainly involve the inhibitory control process. Participants were required to match the color and number with the matching rules to make a decision in the switching task. Therefore, the P3 component may reflect the task decision‐making ability, indicating that the pride emotion inhibits the individual's task decision‐making in the switching task. This indicates that the individual's decision‐making task will be poor under the interference of pride emotion. However, in the inhibitory control task, the participants are required to respond to a small probability stimulus under a high probability background, implying that the participants need to inhibit the dominant response. Therefore, the main response of P3 component may be the inhibitory control. Notably, the P3 difference wave was smaller resulting in a weaker inhibitory control ability. Under the condition of pride, the individual needs to inhibit the dominant response. Moreover, the individual needs to inhibit the effect of pride to complete the inhibitory task. Therefore, the inhibitory control ability of participants in the pride emotion was lower than the neutral emotion. Although the P3 difference waves were different in the two tasks, the findings showed the interference effect of pride on cognitive flexibility and inhibitory control from different aspects.

In summary, the findings from the three experiments conducted in the current study show that pride has different degrees of damage to the three subcomponents of executive function. The damaging effect of pride may be because pride is highly correlated with self and consumes more cognitive resources. Previous studies report that pride is positively correlated with oxyhemoglobin (HbO) level in the blood in the medial prefrontal lobe (mPFC) region. Notably, this region is implicated in self‐related information processing (Hu et al., 2019). In addition, Ding (2018) explored the neural mechanism of pride and reported that pride is an expression of a neural mechanism that points to oneself. Therefore, pride involves more self‐involvement and consumes more cognitive resources compared with basic emotions or neutral emotions. Consumption of cognitive resources implies that resource competition occurs when performing functional tasks leading to a decline in cognitive task performance. The processing efficiency theory proposes that emotions occupy part of the resources of an individual's working memory system (Eysenck & Calvo, 1992). Notably, resources are limited when the remaining resources are insufficient to meet the needs of the task thus leading to a decline in efficiency of cognitive operations. Moreover, Pessoa and Adolphs (2010) proposed the Dual Competition Theory of the interaction between emotion and cognitive processing, which states that emotions and cognitive tasks compete for limited cognitive resources when they are performed at the same time. In addition, DaSilvaet al. (2016) used the implicit association paradigm combined with ERP studies and reported that the late positive slow wave (SPW) amplitude of face recognition in the pride emotion was significantly larger compared with that of the happy emotion. This finding indicates that individuals in the pride emotion require more cognitive resources, thus affecting completion of executive functional tasks.

In addition, impaired executive function under pride emotion can be attributed to the fact that pride is a complex emotional type, which requires involvement of multiple cognitive components. Pride emotion includes several cognitive processes such as self‐evaluation and self‐reflection. The mechanism of pride emotion is more complicated compared with that of basic emotions (Caillaud et al., 2020; Ding, 2018; Sznycer, 2019). The findings showed that the frontal area of the mPFC extending to the dACC was activated under the condition of pride. Furthermore, the ventral prefrontal cortex (vmPFC) extending to the orbital frontal cortex (OFC) brain areas is more active, and these brain areas are toughly correlated with higher‐level cognitive activities such as theory of mind, self‐control, self‐referential processing, and reward processing (Gilead et al., 2016). Therefore, pride which is a complex emotional type related to multiple psychological components requires involvement of various cognitive activities. More resources are required to complete executive function tasks under the condition of such multiple cognitive participation. Therefore, the cognitive activities related to pride and executive function tasks interfere with each other, thus affecting performance of executive function.

These findings show that pride is a unique positive emotion, and may have a significant negative effect on people's cognition. Excessive immersion or pursuit of pride may lead to negative cognitive performance. However, the current study did not subdivide pride. Previous studies proposed a two‐dimensional model of pride (Tracy & Robins, 2004), including authentic pride and hubristic pride. Therefore, further studies should explore effects of the different categories of pride. The findings of the current study provide a basis for further studies on pride emotion implying that when exploring any specific positive emotions, studies should explore it dialectically, accept the multifaceted nature of emotion research, and rationally view the practical significance of research in combination with daily experience.

## CONFLICT OF INTEREST

The author declares no conflicts of interest.

### PEER REVIEW

The peer review history for this article is available at: https://publons.com/publon/10.1002/brb3.2678.

## Data Availability

The data used to support the findings of this study are available from the corresponding author upon request.
